# Workers’ healthy eating practices during the COVID-19 pandemic and their relationship with physical activity and quality of life

**DOI:** 10.1017/jns.2024.45

**Published:** 2024-09-25

**Authors:** Alana do Nascimento Oliveira, Lize Stangarlin-Fiori, Caroline Opolski Medeiros

**Affiliations:** Postgraduate Program in Food and Nutrition, Federal University of Parana, Curitiba, Brazil

**Keywords:** Coronavirus infections, Eating behaviour, Food guides, Health promotion, Nutritional status, Worker’s health

## Abstract

The lifestyle of the population has undergone significant changes due to the COVID-19 pandemic, which could have influenced alterations in dietary habits and overall well-being among workers. This study aimed to evaluate healthy eating practices and their relationship with the workers’ quality of life and physical activity during the COVID-19 pandemic. This was a cross-sectional investigation involving workers in the city of Curitiba, southern Brazil. The study was conducted through the application of an online questionnaire. The data were evaluated using non-parametric tests and fitting a logistic regression model. A total of 123 workers participated in the study, most of them male (53.2%), aged between 31 and 40 years (42.2%), with a predominance of workers with postgraduate degrees (62.6%, *n* = 77), and the majority of workers (68.2%, *n* = 84) were performing their professional activities remotely for at least one day during the week, and 73.2% (*n* = 90). It was observed that 52.8% had excellent healthy eating practices, and the older their age and the greater the practice of physical activity (time and frequency), the better the workers’ healthy eating practices. When assessing quality of life, the lowest average score for healthy eating practices was in the domain of social relationships. A direct relationship of older age, social relationships, and the practice of physical activity with the best individuals’ healthy eating practices was detected. Considering that remote work continues to be adopted post-pandemic, evaluating the dietary practices, physical activity, and quality of life of workers is necessary to understand this new labour phenomenon.

## Graphical abstract

**Figure:**
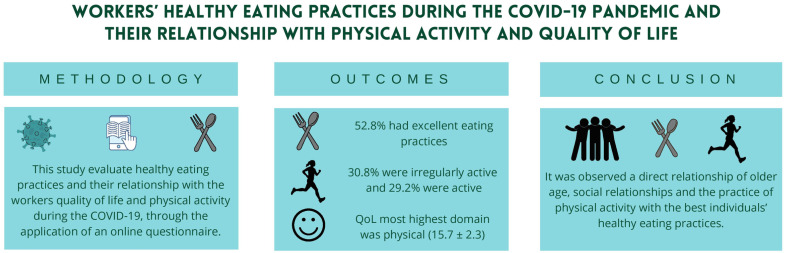

## Introduction

In March 2020, the World Health Organization (WHO) declared a global pandemic due to the new coronavirus (Sars-Cov-2) that causes the COVID-19.^(1)^ By the end of January 2022, more than 356,955,803 cases of COVID-19 and 5,610,291 deaths had been confirmed worldwide.^(2)^ In Brazil, the number of confirmed COVID-19 cases exceeds 24 million, with more than 623 thousand deaths during the same period.^(2)^

During the pandemic, measures such as movement restrictions and lockdowns negatively impacted eating habits and physical activity, leading to negative psychological impacts.^(3)^ Public health measures such as quarantines and social distancing were implemented to contain the virus spread, drastically altering our lifestyle and affecting our physical and mental health.^(3,4)^

It is noteworthy that the main risk factors of COVID-19 are associated with the presence of comorbidities such as Chronic Non-Communicable Diseases (NCDs), which can increase the probability of death, especially in vulnerable groups of all ages.^(5)^

Thus, other important health factors must be addressed, such as diet and physical activity,^(6)^ which may have changed in connection with the new population’s life framework. Thus, the world has experienced two health conditions, coronavirus infections and NCDS increase, and COVID-19 is no longer just a pandemic but is part of a syndemic, that is, a synergy of epidemics.^(7)^

This change in the lifestyle of the population is partly, due to the need for measures to contain the spread of COVID-19, such as social isolation. This has proven to be effective^(8)^ and has led many people to perform their work remotely.^(9)^ This mobility restriction has had direct effects on psychological factors, such as an increase in cases of anxiety and depression and a reduction in the practice of physical activities.^(10–12)^ In addition, eating habits were also influenced both by economic factors, due to the reduction in the population’s income, as well as by the consumption of foods with higher energy density.^(13,14)^

In the period before the pandemic, the consumption of fresh and minimally processed foods represented approximately 70% of the total caloric intake by the Brazilian population, and ultra-processed foods contributed to one-fifth of the calories consumed.^(15)^ Such studies enhance the need for attention to dietary practices, as the consumption of ultra-processed foods tends to have a greater energy density, greater amounts of saturated and *trans* fat, sodium, sugar, and less fibres, which suggests an increased risk of developing obesity and other chronic diseases related to food intake.^(16)^

Changes in eating habits were also observed during the COVID-19 pandemic. Staying at home has also led to the development of unbalanced eating habits The COVID-19 pandemic has resulted in a sedentary life for people under lockdowns and excessive food consumption due to spending much time at home.^(17)^ Due to confinement, some individuals increased the number of meals eaten throughout the day and consumed more snacks between meals.^(18)^ Additionally, an increase in the consumption of ultra-processed products was also observed among people with lower education.^(19)^ On the other hand, other studies did not show changes in the eating patterns of individuals during the pandemic.^(10,13)^

In addition, some individuals improved their diet during this period,^(13,20)^ with a greater consumption of vegetables, fruits, beans, and other legumes.^(19)^ One of the factors that may be associated with this diet improvement is the fear of health problems and the need to stay healthier to face COVID-19.^(13)^ It is also worth noting that due to the pandemic and social isolation measures,^(1)^ food consumption at home increased. This fact, as well as having meals with the family, may have resulted in a positive association to improve the nutritional quality of these meals.^(16)^

Social isolation measures also cause conditions that favour physical inactivity and sedentary behaviour.^(18)^ Yet, the maintenance or improvement of physical activity levels became essential during the pandemic, as it maintains the physiological functions that help to reduce the physical and mental consequences of COVID-19,^(11,21)^ besides contributing to the reduction of anxiety symptoms, improving sleep and the immune system stimulation.^(12)^ For example, Taheri *et al.* ^(3)^ found that increased physical activity during lockdowns was linked to reduced rates of depression, likely because of the beneficial effects of vigorous exercise. The research underscored a link between emotional eating and mood disorders like depression, anxiety, and stress, particularly among elite athletes with decreased physical activity. Emotional eating, prompted by negative emotions such as stress and loneliness, can impact dietary decisions, and vice versa.

Although some studies have assessed the diet^(10,12,13,18,19)^ and some isolated factors such as physical activity^(11,12,20)^ and the individuals’ psychological factors^(10,21)^ during the pandemic, little is known about the association between eating habits and health-promoting actions adopted by people during this period, especially by those who work remotely. Therefore, this study aimed to evaluate the workers’ healthy eating practices and verify their relationship with the quality of life and physical activity of these individuals during the COVID-19 pandemic.

## Materials and methods

### Type of study, sample design and ethics committee

Exploratory, quantitative, descriptive, and cross-sectional study. This study was conducted according to the guidelines laid down in the Declaration of Helsinki and all procedures involving human subjects were approved by the Research Ethics Committee of the Federal University of Paraná (CAAE Nº. 98205318.2.0000.0102). Written informed consent was obtained from all subjects.

The study was conducted online with adult workers (≥ 18 years old) who were allowed to have meals at the Food and Nutrition Units (FNU) of two companies accredited to the Worker’s Food Program (WFP), located in the city of Curitiba, Brazil, which were selected for convenience.

### Data collection

Data collection was carried out during November and December 2020, when the number of COVID-19 cases in Brazil approached 7 million, and deaths reached 200,000, with a daily average of 35,000 new cases.^(22)^

The survey was carried out through an online questionnaire available on the Google Forms® platform that investigated the workers’ description, healthy eating practices,^(23,24)^ physical activity practice,^(25)^ and quality of life.^(26)^ Information regarding the investigation was distributed by e-mail or through telephone contact with the workers. The invited workers were informed about the objectives of the study, voluntary and unpaid participation, preservation of identity, and harmless refusal and/or withdrawal. After reading and agreeing to participate in the survey, the workers answered the questions.

#### Workers’ description

In this section, workers answered questions about age, gender, education, income, eating frequency at the company’s restaurant (FNU), pre-existing medical conditions, consumption of alcoholic beverages and days worked remotely (home office). Information on weight and height were self-reported and the measurements were used to calculate the Body Mass Index (BMI).^(27)^ These variables were structured in the online questionnaire by researchers.

#### Healthy eating practices

The assessment of healthy eating practices was carried out through a questionnaire that is based on the recommendations of the Dietary Guidelines for the Brazilian Population (DGBP)^(23)^ and considers the current healthy eating practices of individuals.^(24)^ This questionnaire validated consists of 24 questions divided into four domains: planning (9 questions), domestic organisation (3 questions), eating modes (5 questions), and food choices (7 questions). The answers to the questions were distributed on a psychometric scale of four options, namely: strongly disagree, disagree, agree, and strongly agree. These answers compose a score from 0 to 72 points and classify the respondents according to their healthy eating practices; the individuals who attained 41–72 points were classified as ‘Excellent’; ‘Keep going’ for those attaining between 31 and 40 points and ‘Attention’ for those who scored 0–30 points.^(16,24)^ The questionnaire assesses current food consumption.

#### Physical activity practice

The level of physical activity practice was measured through the International Physical Activity Questionnaires (IPAQ) short version validated, consisting of eight questions that assess the frequency, intensity, and duration of physical activities reported in the last seven days mat.^(25)^ These activities were divided, according to their intensity, into three categories: walking, moderate physical activity, and vigorous physical activity. After answering the questionnaire, the participants were classified, according to their physical activity practice, as sedentary, irregularly active, active, and very active.^(26)^ Additionally, the frequency and time spent in the practice of physical activity were also considered alone to estimate their association with healthy eating practices.

#### Quality of life

To assess the quality of life, the WHO Quality of Life (WHOQOL-bref) was used, which has 26 questions, consisting of four domains: physical (7 questions), psychological (6 questions), social relations (3 questions), and environment (8 questions).^(28)^ This is a questionnaire validated. In addition, two more questions were evaluated separately, which consisted of the individual’s general perception of quality of life and health.^(28)^ For the assessment, the last two weeks before filling out the questionnaire were taken as a reference. The participants answered the questions according to a psychometric scale of five responses: ‘very good’, ‘good’, ‘not bad nor good’, ‘bad’, and ‘very bad’. When answering the questions, a score was generated according to the domains for each individual.^(29)^

### Data analysis

Data analysis was performed using the Statistica software version 7. To describe the variables observed, descriptive and frequency measures were calculated. Then, to compare the categories of the characterisation variables in relation to the index of healthy eating practices, the Kruskal-Wallis Test was applied, complemented by the Minimum Significant Difference (MSD) test. When the characterisation variable was dichotomous, the Mann-Whitney test was applied. To assess the association between the healthy eating practices index and other quantitative indices, the Spearman’s coefficient was calculated. The same analysis was performed with the classification of healthy eating practices by applying the Chi-square test, when the other variable was categorical, or the Kruskal Wallis test when the variable was quantitative. The lack of normality of the data was confirmed through the Shapiro-Wilk test, which justifies the use of non-parametric tests. To adjust a model that explains the classification of healthy eating practices, logistic regression was used and the Odds Ratio (OR) was identified for the significant variables considering workers’ description, the physical activity practice and the quality of life. Regarding the model’s codes 0 and 1, and 1 represented the excellent level and 0 represented the attention level. The significance limit was considered to be P < 0.05.

## Results

A total of 123 workers participated in the study; most of them were men (53.7%, *n* = 66). There was a predominance of workers with undergraduate and graduate degrees (93.5%, *n* = 115) and 43.1% (*n* = 53) their monthly income was between 2 and 5 minimum wages. In addition, more than half of the workers (52.9%, *n* = 65) were carrying out their professional activities remotely for five or more days during the week, and less than 27.0% (*n* = 33) reported having had meals at the company’s cafeteria during the previous month (Table 1).

**Table 1.** Comparison between the characterisation of workers with the score and categories of healthy eating practices (*n* = 123)

			Healthy eating practices								
					Classification						
	Total		Score	*P* value	Attention		Keep going		Excellent		*P* value
	%	*n*	Mean ± sd		%	*n*	%	*n*	%	*n*	
**General**	100	123	41.0 ± 9.9		13.7	17	33.3	41	52.8	65	
**Gender**				0.58							0.52
Female	46.3	57	40.2 ± 11.6		15.8	9	36.8	21	47.4	27	
Male	53.7	66	41.6 ± 8.4		12.1	8	30.3	20	57.6	38	
**Income**				0.15							0.64
1–2 minimum wages	12.2	15	36.7 ± 9.1		20.0	3	40.0	6	40.0	6	
2–5 minimum wages	43.1	53	39.5 ± 10.7		17.0	9	37.7	20	45.3	24	
5–10 minimum wages	28.5	35	42.8 ± 9.2		14.3	5	28.6	10	57.1	20	
Above 10 minimum wages	8.1	10	43.9 ± 7.5		0.0	0	30.0	3	70.0	7	
Did not answer	8.1	10	46.6 ± 10.4		0.0	0	20.0	2	80.0	8	
**Education**				0.20							0.17
Graduation	30.9	38	39.2 ± 10.9		23.7	9	26.3	10	50.0	19	
Postgraduation	62.6	77	42.3 ± 9.7		9.1	7	35.1	27	55.8	43	
Technical/High School	6.5	8	37.8 ± 6.1		12.5	1	50.0	4	37.5	3	
**Group Age**				0.01*							0.01*
20–30 years	29.3	36	38.9 ± 9.6^b^		13.9	5	47.2	17	38.9	14	
31–40 years	42.2	52	40.3 ± 10.8^b^		21.2	11	32.7	17	46.2	24	
41–50 years	12.2	15	41.1 ± 9.0^ab^		6.7	1	26.7	4	66.7	10	
Above 50 years	16.3	20	46.7 ± 7.0^a^		0.0	0	15.0	3	85.0	17	
**Nutritional status**											
Low weight	1.6	2	55.6 ± 0.7^a^		0.0	0	0.0	0	100.0	2	
Eutrophic	33.3	41	42.2 ± 10.1^a^	0.03*	14.6	6	24.4	10	60.9	25	0.10
Overweight	45.5	56	41.6 ± 9.1^a^		8.9	5	39.3	22	51.8	29	
Obese	19.5	24	36.4 ± 10.5^a^		25.0	6	37.5	9	37.5	9	
**Alcohol consumption**				0,05							0,24
No consumption	39.0	48	38.6 ±10.0		18.7	9	37.5	18	43.7	21	
1–2 d a week	51.2	63	42.5±10.0		11.1	7	28.5	18	60.3	38	
3–4 d a week	8.1	10	39.3±10.5		0.0	0	40.0	4	60.0	6	
5–6 d a week	1.6	2	30.0 ±12.3		50.0	1	50.0	1	0.0	0	
**Meals at the workplace**											0.65
None	73.2	90	40.5 ± 9.9		14.4	13	36.6	33	48.8	44	
1–2 d a week	13.0	16	40.7 ± 10.0		18.7	3	25.0	4	56.3	9	
3–4 d a week	4.1	5	40.2 ± 10.6	0.19	20.0	1	20.0	1	60.0	3	
5–6 d a week	7.3	9	43.1 ± 9.6		0.0	0	33.3	3	66.6	6	
Every day	2.4	3	53.3 ± 10.9		0.0	0	0.0	0	100.0	3	
**Home office/ Remote work**											0.13
None	31.7	39	41.9 ± 10.2		10.2	4	30.7	12	58.9	23	
1–4 d	15.5	19	38.4 ± 10.1	0.44	31.5	6	21.0	4	47.4	9	
5–7 d	52.9	65	41.3 ± 10.3		10.8	7	38.4	25	50.7	33	
**Pre-existing diseases**											
Reported some illness	28.5	35	40.8 ± 9.9	0.98	20.0	7	25.7	9	54.2	19	0.33
Did not report any illness	71.5	88	41.1 ± 10.1		11.3	10	36.7	32	52.2	46	
**Diseases reported**											
Diabetes	3.2	4	42.0 ± 11.6	0.87	0.0	0	50	2	50.0	2	0.63
Hypertension	7.3	9	35.5 ± 10.3	0.08	33.3	3	22.2	2	44.4	4	0.20
Dyslipidemia	8.1	10	43.7 ± 10.5	0.47	20.0	2	20.0	2	60.0	6	0.61
Other (anaemia. gastritis. hypothyroidism. etc)	12.2	17	41.8 ± 9.9	0.48	29.4	5	17.6	3	52.9	9	0.99
**Physical activity**											
Sedentary	13.0	16	33.8 ± 10.4^b^		25.0	4	50.0	8	25.0	4	0.00*
Irregularly active	30.8	38	38.1 ± 10.0^b^	0.00*	23.7	9	36.8	14	39.5	15	
Active	29.2	36	43.0 ± 10.0^a^		11.1	4	27.8	10	61.1	22	
Very active	26.8	33	45.6 ± 10.0^a^		0	0	27.3	9	72.7	24	

Note: Food practice classification- Attention: 0–30 points. Keep going: 31–40 points. Excellent: above 41 points.

P < 0.05* with statistical significance.

Differences not evidenced by MSD test; For the association of the variables regarding the score, Spearman’s coefficient was applied and the Chi-square test for classification; a, b: Different letters indicate the statistical difference.

Most participants (71.5%, *n* = 88) stated they did not have preexisting ailments. The main pre-existing diseases reported, were dyslipidemia (8.1%, *n* = 10), hypertension (7.3%, *n* = 9), and diabetes (3.2%, *n* = 4) (Table 1). Regarding the consumption of alcoholic beverages, almost 61.0% (*n* = 75) of the workers stated that they had drinks at least one day a week (Table 1).

Considering the nutritional status, 65.0% (*n* = 80) of the workers were overweight (overweight or obese) (Table 1). The Kruskal-Wallis test showed a significant difference between the classifications of nutritional status with the mean score referring to the workers’ healthy eating (P = 0.03). However, the MSD test did not confirm this difference. There was also a statistical difference between the age group (P = 0.01) in connection with the workers’ healthy eating practices score. Individuals with and over 41 years of age had an average score of healthy eating practices significantly higher than the group least 41 years of age (Table 1).

Regarding healthy eating practices, in general, the mean score obtained by the total number of participants was 41.0 ± 9.9 (Table 1), and 52.8% (*n* = 65) of the workers had healthy eating practices classified as ‘Excellent’, which represents healthy eating in different aspects according to the DGBP. Then, 33.3% (*n* = 41) had their practices classified as ‘Keep going’, which are those that need to make adjustments in their diet but are moving towards healthy eating. The others had their healthy eating practices classified as ‘Attention’ (13.7%, *n* = 17) and received the lowest score, indicating the need to foster greater changes to make their diet healthier and more adequate to the DGBP recommendations (Table 1).

**Fig. 1.**
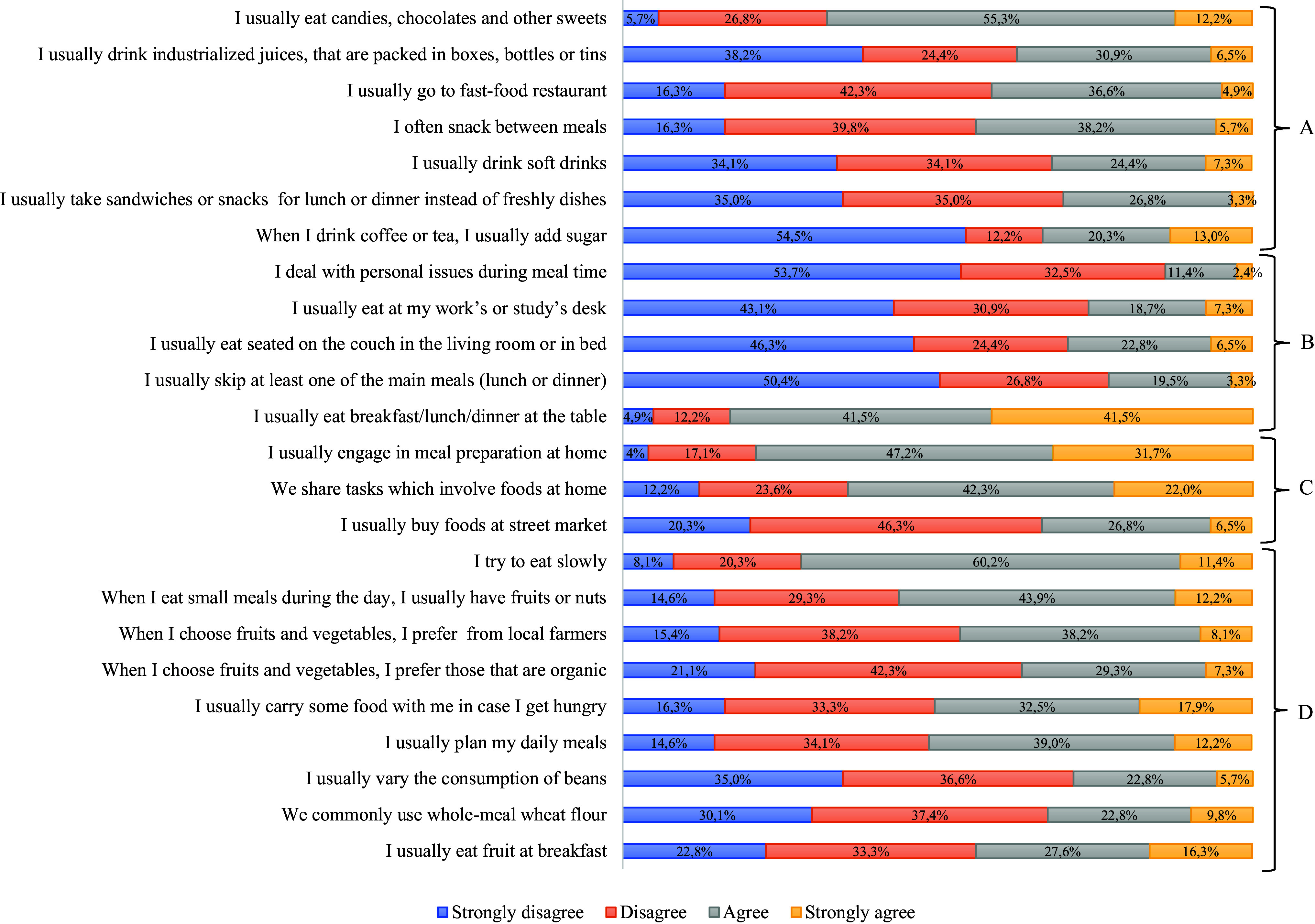
Distribution by percentage of workers’ responses to the questionnaire on healthy eating practices by domains (n=123).

In the distribution of the responses to the healthy eating practices questionnaire with the separation by domains (Fig. 1), it was found that in the domain referring to ‘Food choices’ (Fig. 1a), most participants used to eat candies, chocolates, and others sweets (55.3% agreed), but they were not in the habit of using sugar to sweeten their drinks (54.5% strongly disagreed). In addition, soft drink consumption and the switch of meals for snacks were less common among the participants.

Regarding the ‘Eating modes’ domain (Fig. 1b), the question regarding the practice of having meals sitting at the table was the one that showed the highest adherence, with 83.0% of the participants reporting this practice. Furthermore, the practice of skipping main meals and taking advantage of mealtimes to deal with other things was little practiced among workers, with the responses ‘disagree’ (26.8%) and ‘strongly disagree’ (50.4%) standing out.

As for the domain ‘Domestic organization’ (Fig. 1c), the item with the highest agreement referred to the preparation of food at home, for which the answers ‘agree’ (47.2%) and ‘strongly agree’ (31.7%) were more prominent. The sharing of tasks involving the preparation of food at home was also frequent among the participants (42.3% agreed). In contrast, the purchase of food in street markets was the item with lower adherence, where most responses were ‘disagree’ (46.3%) and ‘strongly disagree’ (20.3%) for this practice.

Regarding the domain ‘Planning’ domain (Fig. 1d), it was found that the habit of varying the pulses in the diet and the consumption of whole wheat flour occurs seldom, as demonstrated by the greater number of ‘disagree’ (36.6%) and ‘strongly disagree’ (30.1%) answers. The purchase of organic products also showed low adherence, with 42.3% disagreeing and 21.1% strongly disagreeing with the statement about this practice. On the other hand, as positive points of this domain, most workers used to take meals calmly (60.2% agreed; 11.4% strongly agreed) and consumed fruits and nuts as snacks (43.9% agreed and 12.2% strongly agreed).

When the level of physical activity practiced by the workers was evaluated, most were irregularly active (30.8%, *n* = 38) and active (29.2%, *n* = 36) (Table 1). According to Spearman’s association coefficient, a significant relationship was identified (P < 0.00), demonstrating that the more active the individual, the better their healthy eating practices (Table 1).

**Fig. 2.**
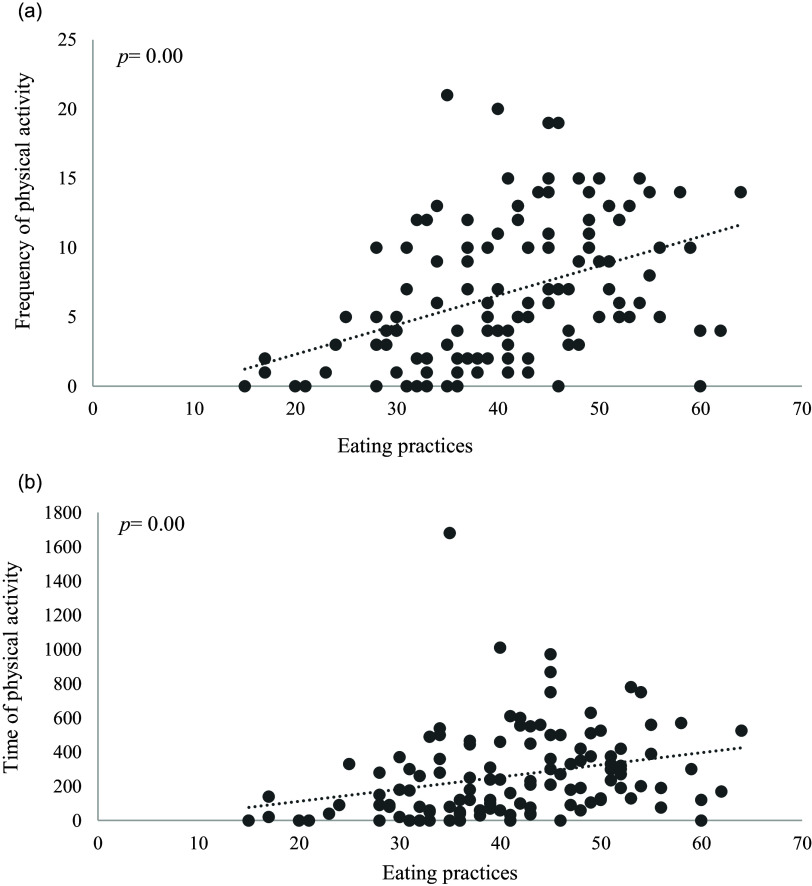
Association between healthy eating practices and physical activity. Note: Statistical significance was accepted as p<0.05 for the association between healthy eating practices and time and frequency of Physical Activity.

The median time spent on physical activities was 190 minutes a week, with a median frequency of 6 times a week. A strong association was found between the classification of healthy eating practices with the frequency of physical activity (Fig. 2a) and with the time spent with the practice of physical activity (Fig. 2b).

When assessing the quality of life of workers, the domain with the highest mean was physical (15.7 ± 2.3), followed by psychological (14.3 ± 2.3) and the one with the lowest mean was social relationships (13.9 ± 2.9) (Table 2). The self-assessment of quality of life had a mean of 14.0 ± 3.6.

**Table 2.** Comparison between the categories of healthy eating practices in relation to the scores of the quality of life domains

Quality of Life Domains	Attention	Keep going	Excellent		General
	(*n* = 17)	(*n* = 41)	(*n* = 65)	P value	(*n* = 123)
	Mean ± sd	Mean ± sd	Mean ± sd		Mean ± sd
Physical	14.70 ± 2.7	15.30 ± 2.4	16.3 ± 1.9	0.03*	15.70 ± 2.3
Psychological	13.00 ± 2.1	13.70 ± 2.5	15.0 ± 2.1	0.00*	14.30 ± 2.3
Social relationships	13.60 ± 3.8	13.30 ± 2.9	14.5 ± 2.5	0.20	13.90 ± 2.9
Environment	13.50 ±1.7	14.26 ± 1.8	15.3 ± 1.8	0.00*	14.70 ± 1.9
QOL self-assessment	11.41 ± 4.2	13.56 ± 3.5	14.9 ± 3.1	0.00*	14.00 ± 3.6

Note: sd = Standard deviation. QOL = Quality of Life. *Statistical significance was accepted as P < 0.05 for the association between QOL domains and the classification of healthy eating practices.

When the assessment of the quality of life was related to healthy eating practices, there was a significant association between the categories of healthy eating practices and the physical (P = 0.03), psychological (P = 0.00), and environment (P = 0,00). Self-assessment of quality of life is also associated (P = 0.00) with healthy eating practices (Table 2).

To identify the strength of the association between the study variables and the classification of workers regarding healthy eating practices, the logistic regression model was applied. The significant variables for the logistic regression model were age, physical activity frequency, and home office work (Table 3). However, no evidence was found when verifying the differences between the home office categories.

**Table 3.** Logistic regression for the classification of healthy eating practices and study variables

Variables	Classification of healthy eating practices		‘Excellent’ Rating		
	Chi square	P value	Estimate	P value	OR
Intercept	0.0	–	–0.043	0.965	
Age	11.6	0.003*	0.749	0.009*	2.11
Nutritional status	2.5	0.28	0.547	0.072	
Physical activity frequency	18.3	0.00*	0.349	0.177	
Physical domain	2.4	0.28	–0.037	0.900	
Psychological domain	2.5	0.28	–0.237	0.496	
Social relations domain	2.4	0.29	0.633	0.046*	1,88
Environment domain	2.3	0.30	0.515	0.085	
Self-assessment domain	2.5	0.27	0.260	0.343	
Gender	1.8	0.40	–0.032	0.928	
Education	4.7	0.31	0.043	0.861	
Wage	12.9	0.11	–0.028	0.917	
Health Problems	2.1	0.33	–0.281	0.430	
Diabetes	3.6	0.16	0.700	0.344	
Hypertension	0.3	0.83	0.064	0.906	
Dyslipidemia	0.0	0.98	0.211	0.693	
Home office	9.9	0.04*	–0.020	0.945	
Meal at work	1.5	0.46	–0.136	0.646	

* Statistical significance was accepted as *P* < 0.05.

When the logistic regression model was applied, two variables were significant in influencing the classification of healthy eating practices at the excellent level (Table 3). The first variable was age, where those aged above the sample mean (44.8 ± 11.2 years) are 2.11 times more likely to achieve an excellent classification in healthy eating practices, compared to those with a lower age (Table 3). The second variable that showed more significance was the social relationships domain of quality of life; in this case, respondents with the highest score in the domain (13.9 ± 2.9) (Table 2) are 1,88 times more likely to achieve an excellent rating in healthy eating practices, in relation to those with lower scores in this domain (Table 3).

## Discussion

The study evaluated the workers’ healthy eating practices and their relationship with the quality of life and physical activity during the COVID-19 pandemic. Workers with and over 41 years of age had better average scores for healthy eating practices. The research demonstrated that people of older age and with better social relationships are more likely to achieve an excellent rating in healthy eating practices. Furthermore, the practice of physical activity frequency is related to the workers’ best healthy eating practices.

### Healthy eating practices

The present study evaluated the adoption of workers’ healthy eating practices during the COVID-19 pandemic. The average healthy eating score was 41.0 ± 9.9, with 52.8% classified as having ‘Excellent’ practices. In specific domains, participants commonly ate sweets but avoided sugary drinks. They often sat down for meals but rarely skipped them or multitasked while eating. Domestic food preparation was preferred over street market purchases. Planning-wise, there was low variation in the consumption of organic products, but there were consumption regular of fruits and nuts as snacks.

It was observed that most participants had their meals at home and were involved in the preparation of meals, which is largely due to the fact that they are performing their work activities remotely (home office). When analysing the workers’ healthy eating practices, using the DGBP recommendations as a parameter, in the domain referring to ‘Planning’, the consumption of fruits or nuts was prominent, a fact that was also identified in another study with Brazilians during the pandemic.^(10)^ This result enhances the idea that some healthy eating practices may have improved during that period. On the other hand, there are also studies indicating that staying at home has led to the development of unbalanced eating habits, resulting in a sedentary lifestyle for people under lockdowns and excessive food consumption due to spending much time at home.^(3,17)^

In addition, in the ‘Planning’ domain, having meals ‘calmly’ also showed high adherence. This statement can be complemented by the habit of having meals sitting at the table, which was highlighted in the ‘Eating modes’ domain. This way of eating during the evaluated period showed that individuals are attentive to the meal period, avoiding performing other activities that interfere with this occasion. In addition, eating behaviours are also influenced by factors such as the place of consumption, which can stimulate stressful situations, leading to the consumption of more energy-dense foods.^(30)^ However, when eating at home, there may be a greater sense of well-being, favouring better healthy eating practices, a fact that occurred more frequently during the pandemic.^(10)^

With regard to preparing meals at home and sharing activities involving food preparation, these were also reported more frequently by workers in the domain of ‘Domestic organization’. This set of healthy eating practices may have benefited from the longer stay of workers in their homes during periods of social isolation, as observed by Coulthard *et al.*,^(31)^ where those who were more involved in the preparation of meals were also able to intake healthier and more adequate food. Low adherence to the purchase of food in street markets was also highlighted in this domain, which may be related to the discontinuation of street market activities, since during the pandemic period some services were discontinued in public places, and were even avoided by the population to prevent the coronavirus contagion.

The domain referring to ‘Food choices’ showed another important factor: the consumption of candies, sweets, and other treats that were greatly appreciated among workers. However, the addition of sugar to beverages and the consumption of soft drinks had low adherence. Data from the Household Budget Surveys (HBS) 2017–2018 already identified that the consumption of soft drinks had decreased among Brazilians.^(15)^ As for the consumption of foods with higher energy density, such as treats and sweet products, this has also been reported in other studies.^(17,31)^ This may be associated with the use of comfort food, related to conditions of anxiety, stress, and depression during the pandemic period.^(32)^ In addition, the prolonged time of social isolation and long remote work routines have also favoured the consumption of these processed foods.^(33)^

The logistic regression model applied in the study demonstrated that age and social relationships are more likely to be related to the workers’ best healthy eating practices. Adherence to national dietary guidelines (DGBP) by older individuals has already been observed in Brazil^(16)^ and France.^(34)^ This may be related to the fact that younger people consume more processed and ultra-processed foods, as these are part of their daily lives since childhood.^(35,36)^ In this younger group, it is already observed that one out of four people between 18 and 34 years of age consumes five or more types of ultra-processed foods, especially margarines and industrialised breads.^(37)^

In addition, young people tend to be less concerned about food quality^(14,38)^ and have greater difficulty with cooking skills.^(39)^ Although this study was carried out during the time of the pandemic, changes in eating habits should be carefully observed, as individual habits tend to be maintained.^(10,13)^ These results enhance the importance of developing good healthy eating practices and practices throughout life, from childhood and adolescence.^(10)^ Thus, the study serves as a warning to pay greater attention to this group in public policies that aim to reduce the consumption of ultra-processed foods.^(37)^ As a result of an unhealthy diet, individuals may become overweight, which is a risk factor for NCDs, responsible for 71% of deaths occurring globally, among adults aged 30–69 years.^(22)^

### Physical activity and healthy eating practices

Just like age, the frequency of physical activity practice was a variable associated with healthy eating practices, where it was observed that the better the eating practices, the greater the practice of physical activity among workers. In the United States, during the pandemic, the practice of physical activity was kept similar to what was current in the previous period; however, a part of the participants (30%) reported having reduced their activity in this period.^(12)^ In a study carried out in the period before the pandemic, with more than 50 thousand adults in Brazil, 44.1% of the population did not reach a sufficient level of physical activity which was less than 150 min per week.^(40)^ The results of the present study show that almost half of the participants needed to improve their physical activity practice during the pandemic.

In order to improve health indicators and encourage the practice of physical activity, the WHO updated its recommendation regarding the time and frequency of physical activity, in 2020, during the pandemic. It was recommended an ideal practice of 150–300 min per week of physical activities, considering those of light or moderate intensity; for intense workouts, the recommendation is 75–150 min per week.^(4)^ This guidance aimed to encourage the practice of physical activity among the population, especially the workers, as a strategy to improve the quality of life and prevent NCDs.^(4)^ There are many reports of the negative psychological impacts related to the COVID-19 pandemic, where restrictions such as home confinement negatively affected levels of physical activity.^(3,41)^

### Quality of life and healthy eating practices

Quality of life assessments showed physical well-being as the highest and social relationships as the lowest. However, as for quality of life, the domain of social relationships was more associated with the workers’ healthy eating practices in the logistic regression model; this conclusion stands out since this was also the domain that exhibited the lowest score. These results demonstrate the potential effects of social isolation, such as the reduction of activities in groups and the imposition of restrictive measures at the state and municipal levels, due to the rules that limited access and permanence in public and private spaces, such as schools, clubs, workplaces, parks, following the WHO recommendations.^(1)^ The emotional support and social network support are important to adherence to a better diet.^(42)^ The DGBP highlights the importance of commensality, as human beings are social beings and having meals with other people favours more suitable environments for food consumption.^(23)^

Also with regard to quality of life, the environment domain was also associated with healthy eating practices, signalling that the environment in which the individual lives can affect their health and individual well-being.^(43)^ Furthermore, the relationships with the environment, when altered, can increase the feeling of insecurity, a fact that occurred during the pandemic due to the fear of contamination by COVID-19.^(32)^ Those individuals who have negative feelings such as insecurity are less willing to improve their diet, while those who have positive feelings are more likely to improve their diet.^(10)^

As observed in the results presented, to promote workers’ health, an approach with different focuses is important in order to improve the workers’ living conditions, avoiding emphasising only weight loss.^(44)^ An adequate work environment can improve the quality of life and help reduce the workers’ NCDs; public authorities’ support is important for these actions.^(45)^ When applied to remote work, specific measures that consider the reality of the worker at home are required.

Some limitations of this research include that due to the high prevalence of COVID-19 disease at the time of the study in the country, it was not possible to use a larger sample and a follow-up test. For this reason, it is suggested that a larger and more diverse sample be used in future research. In addition, the instruments used could be subject to errors as they rely on participants’ memory. However, despite its limitations, the study is innovative, as it is the first to assess healthy eating practices recommended by the DGBP, focusing on workers during the COVID-19 pandemic period. It is further pioneer in verifying the relationship between the healthy eating practices guided by the DGBP with the practice of physical activity and quality of life. Considering that remote work continues to be adopted post-pandemic, future research evaluating the dietary practices, physical activity, and quality of life of workers is necessary to understand this new labour phenomenon.

## Conclusion

Most workers, especially those aged 41 or over and who practiced physical activity, followed the healthy eating practices recommended by the DGBP during the pandemic. The adoption of appropriate healthy eating practices was also strongly associated with the psychological, physical, environmental, and social relationships domains. However, when considering all the study variables, the logistic regression model showed a higher probability of a relationship for the age indicator.

The study also enhances the importance of evaluating the workers’ diet by considering factors such as healthy eating practices, physical activity, and quality of life, in order to guide decision-making and the development of public policies in the framework of promoting health and food and nutritional safety for this population. The study results suggest several implications for public health policies and interventions, such as implementing wellness programmes by companies that encourage healthy behaviours, providing resources for nutritious eating, and promoting physical activity both at work and remotely. Public health campaigns can also educate the population, especially those with pre-existing conditions, about the importance of healthy eating habits, regular physical activity, and lifestyle choices. Additionally, strategies can be developed to integrate health promotion into the workplace culture, including flexible work arrangements that support physical activity and healthy eating habits. Lastly, addressing factors that contribute to overall quality of life, such as social relationships and psychological well-being, can indirectly influence healthy behaviours like eating habits and levels of physical activity.

## Highlights

Most workers had excellent healthy eating practices.

Age was strongly related to the adoption of healthy eating practices.

The older their age, the better the workers’ healthy eating practices.

The greater the practice of physical activity, better the workers’ healthy eating practices.

Adequate healthy eating practices were associated with some quality of life domains.

## Abbreviations

**WHO:** World Health Organization; **NCDs:** Chronic Non-Communicable Diseases; **FNU:** Food and Nutrition Units; **WFP:** Worker’s Food Program; **BMI:** Body Mass Index; **DGBP:** Dietary Guidelines for the Brazilian Population; **IPAQ:** International Physical Activity Questionnaires; **WHOQOL-bref:** World Health Organization Quality of Life; **MSD:** Minimum Significant Difference; **OR:** Odds Ratio; **HBS:** Household Budget Surveys.
